# Supplementary material for: Mechanisms governing the pioneering and redistribution capabilities of the non-classical pioneer PU.1
Source: Nat Commun. 2020 Jan 21;11:402. doi: 10.1038/s41467-019-13960-2 (PMC6972792; doi:10.1038/s41467-019-13960-2)
Supplement: Supplementary file 7 — Source data [file 41467_2019_13960_MOESM7_ESM.zip › Source_Data/FigureS2/FigureS2B_MotifScanOutput/KmeansCluster13/homerResults/motif37.similar.html]

motif37

## Information for motif37

G
T
A
C
C
G
A
T
T
A
G
C
T
A
C
G
A
G
T
C
C
T
A
G
T
C
G
A
A
C
G
T
G
T
C
A
G
C
A
T
  
Reverse Opposite:  

C
G
T
A
C
A
G
T
T
G
C
A
A
G
C
T
G
A
T
C
A
C
T
G
A
T
G
C
A
T
C
G
G
C
T
A
C
A
T
G
  

|  |  |
| --- | --- |
| p-value: | 1e-17 |
| log p-value: | -4.001e+01 |
| Information Content per bp: | 1.613 |
| Number of Target Sequences with motif | 59.0 |
| Percentage of Target Sequences with motif | 3.49% |
| Number of Background Sequences with motif | 378.5 |
| Percentage of Background Sequences with motif | 0.84% |
| Average Position of motif in Targets | 104.6 +/- 54.2bp |
| Average Position of motif in Background | 99.3 +/- 71.9bp |
| Strand Bias (log2 ratio + to - strand density) | -0.1 |
| Multiplicity (# of sites on avg that occur together) | 1.12 |
| Motif File: | file (matrix) reverse opposite |

### Similar de novo motifs found

|  |  |  |  |  |  |  |  |
| --- | --- | --- | --- | --- | --- | --- | --- |
| Rank | Match Score | Redundant Motif | P-value | log P-value | % of Targets | % of Background | Motif file |
| 1 | 0.812 | C T G A T C G A T C G A A G C T G T A C A G C T A T G C A C T G A G T C A T C G G C T A A C T G T C G A G C A T | 1e-15 | -36.777261 | 1.71% | 0.20% | motif file (matrix) |
| 2 | 0.894 | G C A T T G A C C A G T A G T C A C T G A G T C A C T G C T G A C A T G T C G A C G A T G A T C | 1e-15 | -35.919480 | 1.83% | 0.24% | motif file (matrix) |
| 3 | 0.826 | C G A T G T A C A C G T A T G C A C T G A G T C A C T G T C G A C A T G G C T A C A G T G A C T A G C T | 1e-14 | -33.452001 | 1.18% | 0.08% | motif file (matrix) |
| 4 | 0.810 | C A G T G T A C A C G T T A G C A T C G A T G C A C T G T G C A A C T G G C T A A G C T | 1e-14 | -32.441763 | 1.48% | 0.16% | motif file (matrix) |
